# Genetic validation of whole-transcriptome sequencing for mapping expression affected by *cis*-regulatory variation

**DOI:** 10.1186/1471-2164-11-473

**Published:** 2010-08-13

**Authors:** Tomas Babak, Philip Garrett-Engele, Christopher D Armour, Christopher K Raymond, Mark P Keller, Ronghua Chen, Carol A Rohl, Jason M Johnson, Alan D Attie, Hunter B Fraser, Eric E Schadt

**Affiliations:** 1 Merck Research Laboratories, Research Informatics, 33 Avenue Louis Pasteur, Boston, MA, 02115, USA; 2 Rosetta Inpharmatics, LLC, a wholly owned subsidiary of Merck & Co., Inc., 401 Terry Ave N, Seattle, WA, 98109, USA; 3 University of Wisconsin-Madison, Department of Biochemistry, 433 Babcock Drive, Madison, WI, 53706, USA; 4 Currently at Department of Biology, Stanford University, 371 Serra Mall, Stanford, CA, 94305, USA; 5 Currently at Pacific Biosciences, 1505 Adams Drive, Menlo Park, CA, 94025, USA; 6 Currently at NuGEN Technologies, 201 Industrial Road, San Carlos, CA 94070, USA

## Abstract

**Background:**

Identifying associations between genotypes and gene expression levels using microarrays has enabled systematic interrogation of regulatory variation underlying complex phenotypes. This approach has vast potential for functional characterization of disease states, but its prohibitive cost, given hundreds to thousands of individual samples from populations have to be genotyped and expression profiled, has limited its widespread application.

**Results:**

Here we demonstrate that genomic regions with allele-specific expression (ASE) detected by sequencing cDNA are highly enriched for *cis-*acting expression quantitative trait loci (*cis-*eQTL) identified by profiling of 500 animals in parallel, with up to 90% agreement on the allele that is preferentially expressed. We also observed widespread noncoding and antisense ASE and identified several allele-specific alternative splicing variants.

**Conclusion:**

Monitoring ASE by sequencing cDNA from as little as one sample is a practical alternative to expression genetics for mapping *cis*-acting variation that regulates RNA transcription and processing.

## Background

The genetics of genome-wide gene expression has emerged as an important new field with potential to transform our understanding of a broad scope of topics, ranging from basic regulation of transcription to mechanisms of complex human diseases. In most studies of gene expression genetics, genetically diverse individuals are genotyped at genetic markers that characterize most of the common DNA variation in the population and are also phenotyped by measuring the abundances of thousands of mRNA transcripts [1]. These molecular phenotypes are then genetically mapped like any other quantitative trait, revealing quantitative trait loci (QTL) where a polymorphism affects a transcript's abundance levels [1]. These studies have led to the construction of regulatory networks that are predictive of disease states such as obesity in both mouse and human [2,3]. They have also shown that gene expression QTL (eQTL) in humans can uncover the mechanisms of action of disease-associated SNPs previously implicated by genome-wide association studies (GWAS) [4,5], and have even implicated new SNPs as additional disease-associated loci [4]. At the heart of all eQTL studies is polymorphic gene expression. This can be caused by genetic variants that act in *cis *or in *trans*. A major difference between the two classes is that because a *cis*-acting allele acts only on the chromosomal copy on which it resides, a heterozygous *cis*-acting polymorphism results in allele-specific effects, such as higher expression of allele A vs allele B, even though both alleles are present in the same nucleus and thus experience the same *trans-*acting environment [6]. In contrast, a heterozygous *trans-*acting polymorphism cannot lead to allele-specific effects, because it does not differentiate between the two alleles.

Most studies of gene expression genetics have classified expression variation within species as due to either *cis*- or *trans-*acting factors; this is typically done by genetically mapping variation in expression levels, and inferring that a gene whose expression maps very close to the gene itself is most likely influenced by one or more *cis*-acting variants [1,6]. *Cis*-acting polymorphisms have been found to be extremely common in all studies published to date, and generally exert much stronger effects on gene expression than do *trans-*acting polymorphisms. For example, recent studies of human gene expression genetics have inferred between 85-100% of significant eQTLs to be *cis*-acting [2,5], although our power to detect these two types of events has not been exhaustively compared. *trans-*acting eQTL may eventually be found to be more prevalent, once larger sample sizes and thus greater power are achieved, and they have also been found to be useful in the context of coexpression networks and causal inference [2,3,5].

It has been appreciated for many years--since well before the first studies of genome-wide gene expression genetics--that measuring ASE can reveal the presence of heterozygous *cis*-acting polymorphisms. In fact, ASE is a necessary consequence of heterozygous *cis*-acting polymorphisms. ASE can be measured by quantifying the abundance of each allele of a transcribed heterozygous polymorphism (such as a SNP) among a gene's transcripts. Since the genomic ratio of heterozygous alleles in a diploid is 1:1, any significant deviation from this among mRNA transcripts suggests allele-specificity. Primarily due to technical limitations, measuring ASE has not been widely applied to identify *cis*-acting polymorphisms. The emergence of high-throughput cDNA sequencing (i.e. RNA/NSR-seq) has enabled whole-genome identification of ASE and has been applied to mapping imprinted loci [7,8]. In addition, two targeted approaches employing PCR [9] and padlock-capture [10,11] have shown that up to 25% of genes may be preferentially expressed from one allele and that a significant proportion of these are tissue-specific [11].

Although it is assumed that identifying ASE by high-throughput sequencing is an alternative to mapping *cis*-eQTL by microarrays, this has not been shown experimentally. *cis*-eQTL result in higher expression of one allele over the other in any given sample. This bias consistently favors one parental allele if the causal polymorphism is in linkage disequilibrium (LD) with the transcribed SNPs used to quantify ASE. In this study, we validate cDNA sequencing toward identifying regions affected by inheritable *cis*-acting regulatory variants by demonstrating extensive overlap between ASE and *cis*-eQTL.

## Results

### Mapping *cis*-eQTL

Using a custom Agilent murine microarray [12], we profiled 500 adipose and islet samples from an F2 intercross population constructed from the BTBR and C57BL/6J strains on an Ob null background (referred to here as the BTBRxB6 cross), and detected *cis*-eQTL for expression traits using a standard regression procedure [12,13]. We excluded 1,291 genes from this analysis in which probes overlapped a known [14] or predicted (see Methods) BTBR/B6 SNP from our analysis, since these can lead to false-positive *cis*-eQTL by impacting hybridization kinetics. At LOD > 3 (FDR = 0.01) we detected 3,367 and 3,819 *cis*-eQTL genes (of 34,257 on array) in adipose and islets respectively which we used as baseline lists for comparison.

### Mapping ASE

**Figure 1:**
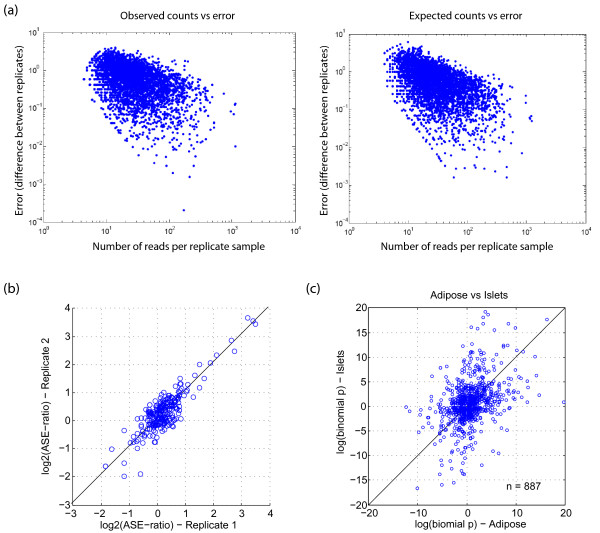
**Reproducibility and tissue specificity of ASE measured with NSR-seq**. **(a) **Observed and expected error in biological replicates. RNA was isolated from independent samples, and subjected to NSR-seq (see methods). The error was calculated as the difference in log2(allelic ratio) between the two replicates, and is shown in relation to the average number of reads for the SNP in the two replicates (left panel). As expected, SNPs with more reads show lower error. The theoretically expected errors from the binomial distribution (right panel). Simulated data was not significantly different than the real data, when comparing errors by the Wilcoxon test (p > 0.2). **(b) **Ratio of allele-specific sequencing reads between two biological replicates for RefSeq genes with at least 100 reads. *R*^2 ^= 0.84 (n = 289, p = 2.4e-115) **(c) **ASE conservation of RefSeq genes between islets and adipose. log(binomial-p) reflects confidence of ASE, and was arbitrarily set to negative when bias was toward B6 allele. *R*^2 ^= 0.20 (n = 887, p = 2.4e-115)

We performed NSR-seq [15] on 100 pooled adipose and 100 pooled islet samples randomly chosen from the F2 samples (above). We also performed NSR-seq on 100 pooled liver and 100 pooled hypothalamus samples to assess degree of ASE conservation across tissues (but did not profile these on arrays). As described previously [7-11], we identified ASE by taking advantage of sequencing reads that overlap SNPs, using the base identity to discriminate allelic origin. More than 1,500 genes were represented by at least 10 allele-specific sequencing reads (i.e. overlap a BTBR/B6 SNP) in islets and adipose (Additional File 1, Figure S1), which we empirically found to be a practical minimum for identifying ASE. We assessed the probability of ASE using the cumulative binomial distribution since it models the expected number of counts of two types, each with a certain probability of occurrence. For example, flips of a fair coin follow the binomial distribution, with probability of 0.5 for heads or tails in each flip. Similarly, allele-specific read counts can be regarded as independent trials, where each allele has some probability of being observed (0.5 for autosomal alleles in F1 s, genotype frequency of pool in F2s). In order to assess the accuracy of using the binomial expectation to estimate the significance of deviation from random, we analyzed allelic expression of biological replicates from a previous study in which we studied ASE by NSR-seq in mouse embryos [7]. We found no significant difference in the extent of agreement between allele-specific read counts of two independently generated NSR-seq libraries compared to randomly generated counts from the binomial distribution (Figure 1a), indicating that our data can be accurately modeled by this distribution. We also observed good agreement in allelic ratios between these two biological replicates (Figure 1b). Finally, in agreement with Emilsson et al. [3] and Zhang et al. [11], in pairwise comparisons of ASE across four tissues we observed general agreement in direction of bias toward the same strain, suggesting that *cis*-acting effects tend to be conserved across tissues/cell-types (Figure 1c; Additional File 1, Figure S2).

Under the null-hypothesis (no allelic bias), the ratio of BTBR to B6 reads reflects the allelic proportions within the RNA pool. Although the average allelic ratio was 50:50, we used the microarray genotyping information to augment the precision of our analysis by exactly defining the ratio at each locus (see Methods). 2,230 adipose and 1,444 islets genes met our criteria for reliably measuring ASE, most notably containing a minimum of 10 allele-specific sequencing reads covering at least three SNPs (see Methods). 719 and 501 genes respectively were also detected in *cis*-eQTL which by itself represents a significant overlap (Fisher Exact Test p < 1e-11).

### ASE identified by NSR-seq agrees with *cis*-eQTL identified by microarrays

**Figure 2:**
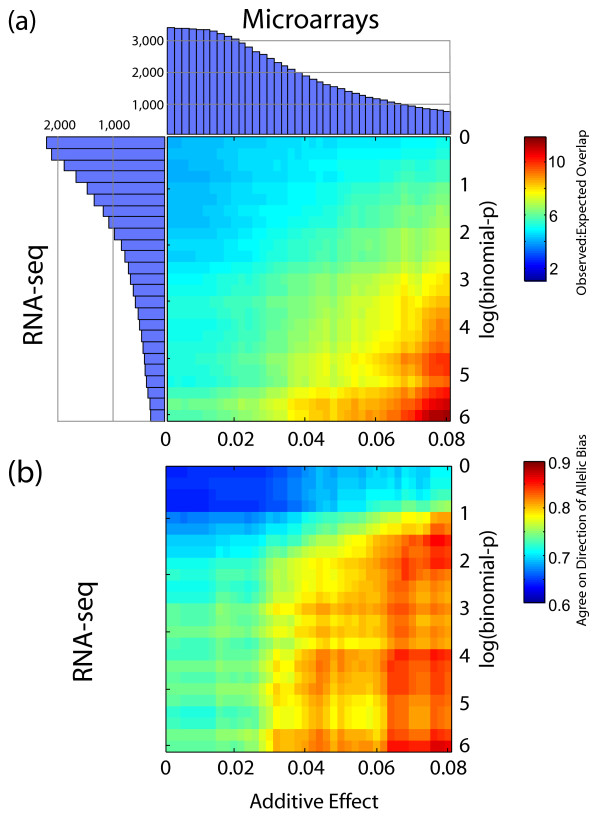
**Genes mapped to *cis*-eQTL (Array) and genes under allele-specific expression (ASE; NSR-seq) overlap and agree on direction of allelic bias in adipose**. **(a) **Ratio of observed to expected level of overlapping genes exceeding Genetic Additive Effect and ASE confidence scores. Histograms along the axes depict the number of genes exceeding Additive Effect or binomial-p thresholds. **(b) **Proportion of overlapping genes for which ASE and Additive Effect agree on direction of allelic bias (i.e. higher expression detected from B6 or BTBR allele by both approaches).

We observed a significant overlaps between *cis*-eQTL genes and ASE genes, and the overlap improved with increasing confidence thresholds of each method (Figure 2). We used genetic additive effect to quantify *cis*-eQTL since it captures the magnitude of the difference in transcriptional abundance across the three possible genotypes at each marker, and all *cis*-acting eQTL are expected to show additive behavior. In adipose, the ratio of observed to expected (see Methods) gene overlap exceeds 10 at high thresholds (Figure 2a). Since the overlap is in part dependent on expression (eQTL and ASE are easier to detect for highly expressed genes), we also assessed agreement on the direction of bias (B6 vs BTBR; Figure 2b). Again, agreement improved with increasing thresholds, frequently exceeding 80% (e.g. at LBP > 2, add. eff. > 0.05, 115 genes were detected by both technologies, of which 92 agreed on direction of bias). We observed similar trends in islets with marginally lower levels of enrichment (Additional File 1, Figure S3), possibly due to increased variance resulting from fewer expressed genes and fewer genes exhibiting ASE (Additional File 1, Figure S1).

Previous sequencing-based ASE studies used F1 s [7,8] where both copies of each allele are always present at exactly equal ratios, thus enabling a simple statistical analysis of ASE. Our primary goal was validation of the method. Using the same samples for identifying both ASE and *cis*-eQTL was thus essential for avoiding inter-sample biases and additional effects induced by the exceptionally high heterozygosity of F1 s. Nonetheless, we observed strong overlaps in F1 s as well (Additional File 1, Figure S4), demonstrating that using single samples is sufficient to map regions affected by *cis*-acting variation and that artificial F1 effects are not extensive.

A number of explanations may account for any disagreement on the direction of bias between the NSR-seq and microarray data. Most obvious are the technical differences in determining transcriptional abundance: microarrays monitor 3'UTR abundance whereas NSR-seq captures the entire transcript. Any transcriptional processing effect not reflected in the 3'UTR may thus lead to an overall difference, and any 3'UTR processing effect will be exaggerated on arrays. Furthermore, SNPs in microarray probe regions may lead to artifactual differences in BTBR and B6 transcript level measurements, and false-positive SNP predictions that artifactually bias sequencing allele counts toward B6 (the reference genome). To gain insight into these discrepancies, we Sanger-resequenced SNPs in all genes that disagreed in their direction of bias between the two technologies at high ASE and *cis*-eQTL thresholds in adipose (n = 25 adipose samples, |log(binomial-p)| > 3, |add. eff.| > 0.05). Strain bias in 7 genes could not be sufficiently distinguished by analyzing trace files. Of the remaining 18 genes, 15 (83%) agreed in direction with NSR-seq, of which 5 (28%) had no detectable BTBR signal, presumably due to a false-positive SNP, and 3 (17%) agreed with microarray-inferred strain bias (Additional File 1, Table S1). This suggests that measuring ASE by high-throughput sequencing may be less susceptible to artifacts than mapping cis-eQTLs in an F2 population.

### ASE is widespread and encompasses noncoding RNA

**Figure 3:**
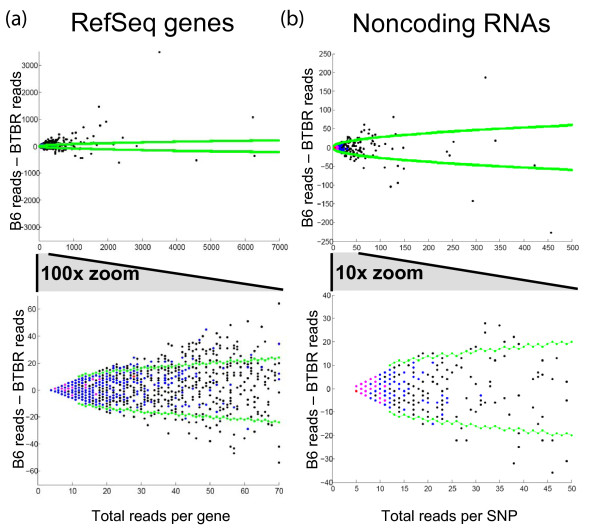
**Visualization of allelic bias in NSR-seq data**. **(a) **For each RefSeq transcript in adipose, the total number of informative reads was plotted against the difference in reads between alleles. The 99% confidence interval (from the binomial distribution) is shown in green, with points above the upper line or below the lower line falling outside the expected range for 99% of a random data set (lacking ASE). **(b) **The same as part **(a)**, but showing SNPs that fall outside of RefSeq transcripts. These regions were not assayed on our microarrays, but still show pronounced ASE.

Since NSR-seq captures all transcripts regardless of polyadenylation state [15], we were able to assess ASE across both coding and noncoding regions. In agreement with previous reports [9-11,16,17] we identified extensive ASE among coding genes (Figure 3a; adipose shown, see Additional File 1, Figure S5 for islets). We also observed higher than expected levels of ASE in noncoding genes (Figure 3b; genes include tRNA, snRNA, snoRNA, miRNA precursors and other smaller classes of RNA compiled from fRNAdb [18]). In these plots, points falling near zero on the Y-axis indicate a lack of allele-specificity. Using binomial statistics as described above, we also plotted the 99% confidence intervals as a function of the total number of informative reads per transcript (Figure 3, green lines). Many genes fall outside of the 99% binomial confidence limits (much greater than 1%), indicating that allele-specific expression is widespread in these mice.

Comparing the complete observed and expected distributions of allele-specificity (see Methods), we found that 36.7% of the BTBR SNPs covered by at least 10 reads in adipose showed greater allele-specificity than expected, and at 100× coverage, this increased to 59.9%. In the islet data, allelic bias was even more common: 42.0% of SNPs were biased at 10× coverage, and 68.0% were biased at 100×. At 100× coverage, we can detect 1.70-fold differences at p = 0.01 with 50% power (or at p = 0.05 with 82% power); at 1000× coverage, this drops to 1.18-fold at p = 0.01 with 50% power (or 78% power at p = 0.05). Unfortunately we cannot estimate the total extent of ASE, since this strongly depends on the distribution of effect sizes for *cis*-eQTL (e.g., having many weak effect sizes below our current detection threshold would indicate there is still much more ASE to be found). Future studies with higher coverage of the transcriptome will be able to address this issue.

### Antisense transcription occurs more frequently from the same allele vs. independent alleles

Widespread antisense transcription in mammals is well documented [19-21], although a general function for these transcripts has not been established. Recently, several groups have identified clustering of short antisense transcripts immediately upstream of transcription start sites (TSSs) [22,23]. In yeast, these short transcripts are immediate targets of the exosome suggesting that they are non-functional derivatives of an intrinsically bidirectional RNA polymerase II [24]. The strand-specific nature of our RNA amplification protocol allowed us to further explore this by further dissecting antisense transcription by their allelic origin. In order to maximize the number of assayable sites with antisense transcription, we used previously published data from F1 B6xCAST/Ei embryos [7], which have on average >4-fold higher SNP density over B6/BTBR [14]. We have also done these analyses with BTBRxB6 data and achieved similar, but as expected, weaker trends resulting from lower sensitivity (BTBRxB6 analyses shown in Additional File 1, Figure S6).

**Figure 4:**
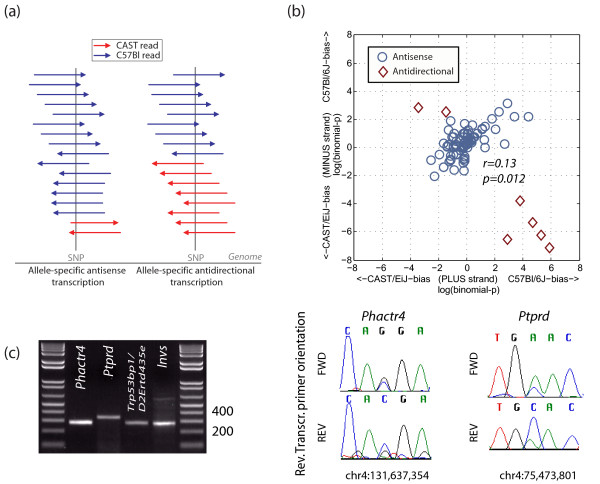
**Antisense transcription from same allele is more common than antidirectional transcription from separate alleles**. **(a) **Schematic distinguishing these two possibilities. **(b) **LBP scores of antisense expressed SNPs as a function of genomic strand. Points along positively sloping diagonal correspond to antisense expression from the same allele, points along negatively sloping diagonal (red diamonds) correspond to antidirectional allele-specific expression. Diamonds correspond to validated antidirectional events. **(c) **Validation of allele-specific antidirectional transcription of top four candidates (three of the SNPs are from same gene). In all cases, direction of bias agreed with NSR-seq. Genomic coordinates correspond to genome release NCBI build 36.

Allele-specific antisense transcription occurs in two forms: antidirectional, where transcription is restricted to separate alleles and occurs in only one direction from each allele (Figure 4a), and allele-specific antisense, where both directions are transcribed from one allele, and little or no transcription occurs on the other (Figure 4a). Since antidirectional transcripts require independent *cis*-acting regulation, predominance of these events over allele-specific antisense transcripts would suggest that antisense transcription in general is a functionally regulated process. Although we observed both types of events, 65/106 of SNPs with bidirectional transcription (|LBP| > 0) were biased toward expression from the same allele, versus separate alleles (p = 0.0125, Figure 4b). Of seven SNPs with the strongest antidirectional transcription (red diamonds in Figure 4b), four are within introns of *Phactr4*, one is intronic to *Ptprd*, and the final two are located in overlapping antisense UTRs (*Trp53bp1*/AK050250, and *Invs*/BC006867). We validated allelically biased bidirectional transcription in all four of these regions by Sanger-sequencing (Figure 4c; and see Additional File 1, Figure S7). Although our power to make conclusive claims is low, we observed a significant correlation between the strand of transcription and allelic bias (i.e. allelically-biased antisense transcription tends to occur from the same allele). This is in agreement with the idea that antisense transcription is in general not an independently regulated process but rather a consequence of coding-gene expression.

### Identification of allele-specific splicing events

Pre-mRNA splicing is a highly regulated process that involves interplay among dozens of regulatory factors and RNA sequence motifs. The importance of this process is epitomized by estimates attributing 15-50% of human genetic diseases on mutations in splice-site regulatory sequences [25]. Although these *cis*-acting effects are widely thought to exist, they are difficult to detect systematically [4,26]. By counting sequencing reads that map over splice junctions and a SNP, NSR/RNA-seq is uniquely suited to detect these events. This approach is theoretically possible for any reads that are spliced and overlap a SNP, but is complicated by differences arising from allele-specific transcription. We controlled for this effect by focusing on SNPs that overlap alternatively spliced reads, searching for cases where the skipped isoform has an allelically shifted ratio compared to the included isoform (Additional File 1, Figure S8). We report 10 candidate examples (Table 1). In most cases the skipped isoform is predicted to disrupt a conserved protein domain, suggesting that even minor allele-specific splicing changes may have dramatic functional effects.

**Table 1:** Allele-specific splicing candidates.

Gene	Exon Included Reads (B6:CAST)	Exon Skipped Reads (B6:CAST)	p(ASE) is less than*	Gene function (Gene Ontology)	Conserved protein domain encoded by skipped exon (Pfam)
Slc8a1	6 (2:4)	429 (232:197)	0.344	reduction of cytosolic calcium ion concentration	No domain reported
Baz2b	30 (9:21)	99 (58:41)	0.021	chromatin modification	Bromodomain adj. to zinc finger domain (PTHR22880)
Eif4enif1	24 (3:21)	18 (12:6)	0.119	protein transport	No domain reported
Epb4.1l2	7 (5:2)	6 (2:4)	0.344	cortical actin cytoskeleton organization	4.1 C-terminal domain (PF05902)
Papd4	6 (6:0)	3 (0:3)	0.125	polynucleotide adenylyltransferase activity	PAP/OAS1 substrate-binding domain (SSF81631))
Aqr	8 (8:0)	3 (0:3)	0.125	RNA splicing; body morphogenesis	DNA2/NAM7 helicase family member (PTHR10887:SF5)
Ankrd12	32 (9:23)	3 (3:0)	0.125	unknown	Ankyrin repeat (SSF48403)
Nudc	27 (11:16)	75 (75:0)	0.221	nuclear migration; nervous system development	CS (PS51203), HSP20-like chaperones (SSF49764)
Rbm17	32 (16:16)	5 (5:0)	0.031	mRNA processing, RNA binding	Splicing factor 45 (PTHR13288:SF9)
Cacna1h	1118 (378:740)	5 (5:0)	0.031	Calcium transport	Voltage-gated_potassium_channels (SSF81324)

## Discussion

We found a highly significant overlap in *cis*-eQTL genes identified by microarray profiling and ASE genes identified by NSR-seq. Improving overlap with increasing additive QTL effect sizes and/or LBP demonstrates that, as expected, both approaches reliably detect allele-specific expression despite a number of key differences in the technologies. For example, the majority of microarray gene-expression platforms, including the platform used here, rely on 3'-biased amplification protocols and thus position microarray probes near the 3'-end of the gene. With NSR-seq we monitored the entire transcript, including introns, which we previously found to improve sensitivity [7] presumably because most intronic reads correspond to unprocessed pre-mRNA or degradation products. Any allele-specific events outside the microarray probe region will thus skew the comparison. Unknown SNPs can also cause disagreement: 1) they can lead to artifactual genetic associations if they are within probe regions [6], 2) can bias allelic representation in NSR-seq if located within priming sites, and 3) can lead to a bias towards aligning NSR-seq reads to the reference genome (though this last scenario is expected to be rare, since it requires having both a known and an unknown SNP in the same read; consistent with this expectation, visual inspection of Figure 3 reveals little bias). False-positive SNPs accounted for 28% of extreme cases where NSR-seq disagreed with microarrays. Although this is a substantial overestimate of total effect since selection of these genes was biased toward strong disagreement, it highlights the importance of high-quality SNP maps for both methods.

While our results mostly agree with those from more traditional approaches using hundreds of microarrays to measure gene expression among F2 mice, there are several distinct advantages of the NSR-seq approach. First, NSR-seq results are not limited by a fixed set of probes, and because of this we were able to find allele-specific instances of splicing and antisense transcription that were invisible to our microarrays. Second, identification of *cis-*eQTL does not depend on arbitrary genomic distance cutoffs with NSR-seq, in contrast to microarray studies which will inevitably misclassify some *trans*-eQTL as *cis*, and *vice versa*. Third, the NSR-seq approach can be applied to any outbred diploid or polyploid species, even those for which microarrays are not readily available. Finally, NSR-seq can be applied to a single F1 individual, with a single sequencing run costing several thousand dollars, as opposed to applying microarrays to each of hundreds of F2 individuals, saving a great deal of time and expense (though pooling F2 individuals may more closely agree with *cis*-eQTL from microarrays, if genetic interactions in the F2 population are not captured in the F1). On the other hand, the greatest disadvantage of NSR-seq is its inability to detect *trans*-eQTL. Measuring ASE in the context applied here where animals were pooled at the RNA level cannot be used to map the genomic region that contains the causal effect. Sequencing or microarray profiling many samples will always be required for mapping QTL. A second disadvantage is that in outbred species with short LD blocks (such as human), the causal *cis*-eQTL polymorphism will often not be in LD with any transcribed SNPs, and in these cases pooling will not reveal ASE. Third, pooling RNA from different individuals could introduce biases. These can be minimized by keeping track of the mass of RNA from each individual or by pooling by equal mass. It is also possible that one or a few individuals in the pool have significantly different expression levels and contribute unbiasly to the ASE signal. We show that this is very unlikely given our strong agreement on ASE in F1 biological replicates (Figure 1a), but ruling it out for the entire pool would require testing individuals. Nevertheless, pooling human samples has already been shown to reveal many *cis*-eQTL [17]. To detect *cis*-eQTL where the causal polymorphism is not in LD with any transcribed SNPs, NSR-seq can be performed on individual samples, as long as genotype phasing is known [17]. A method able to quickly and efficiently identify *cis*-eQTL genome-wide will find applications in many areas. The dependence of *cis*-eQTL on environmental conditions--a subject not previously studied, in large part due to its prohibitive cost--can now be studied efficiently and comprehensively. The method can be applied to hybrids between distinct species (as has already been done with low-throughput pyrosequencing for *Drosophila *hybrids) to reveal all *cis*-acting gene expression differences, and inform us of the importance of *cis*-regulation in evolution. Since the action of positive selection can now be inferred solely from *cis*-eQTL (H. Fraser; personal communication), selection on gene expression can be measured by NSR/RNA-seq in a wide range of species. And finally, *cis*-acting polymorphisms have been shown to be highly enriched for SNPs associated with human disease risk in genome-wide association studies, so compiling catalogs of genes affected by *cis*-eQTL in various tissues, populations, and disease states could be extremely useful for inferring which disease associations are likely due to *cis*-acting effects on gene expression, and even more importantly, which genes are perturbed by the disease-associated variants (see Additional File 1, Supplement).

## Conclusions

Validated against expression genetics, cDNA sequencing is an effective strategy for identifying allele-specific expression which can be used to map inheritable cis-acting variation. Application across multiple samples has potential to yield insight into polygenic phenotypes including complex disease.

## Methods

### Sample collection and microarray analysis

The BTBR × B6 F2 mice were constructed by intercrossing F1 animals obtained by crossing C57BL/6 (B6) BTBR mice carrying the leptinob/ob (ob) mutation. The resulting F2 animals were housed in an environmentally-controlled facility on a 12 hr light/dark cycle (6 AM - 6 PM, respectively). Mice were provided free access to water at all times and to a standard rodent chow (Purina #5008) *ad libitum*, except during a fasting period (8 AM - noon) in order to obtain plasma at 10 weeks of age, after which they were sacrificed by decapitation. For each animal the right gonadal fat pad (adipose) and pancreas were collected for expression profiling. The adipose tissues were flash frozen in liquid nitrogen. Intact pancreatic islets were isolated from the F2 mice using a collagenase digestion procedure as previously described [27]. A detailed description of islet isolation, RNA purification, and microarray analyses is available in Additional File 1 (Supplementary Methods). All animal handling procedures were approved by University of Wisconsin Animal Care and Use Committee.

### NSR-seq

Total RNA from 100 adipose, islet, liver, and hypothalamus samples (see above) was pooled and subjected to strand-specific, whole-cell NSR-seq [15]. Libraries were sent to Illumina (Hayward, California) for single-end 36 nt sequencing for total depth of 2 G/sample. Novoalign (Novocraft) was used to align against NCBI mouse genome release 36 (UCSC Feb. 2006 release [mm8]) and a collection of splice junctions generated from Refseq genes [28], ENSEMBL genes [29], and UCSC Known Genes [30]. Predicted splice junctions from ESTs [30], Genscan [30], and N-scan predictions [30] were also considered in regions that lack coding gene models. All possible splice sites spanning up to two exon skipping events in gene/transcript models above were represented. A minimum of 5 nt overlap per flanking junction sequence was required for alignment to be considered, selected on basis of maximizing overall alignment sensitivity (data not shown). All reads that aligned uniquely to the genome or splice-sites, and redundantly mapped reads that overlap unique reads in only one genomic location, were retained for further analysis. 73,556,741/89,349,136 adipose, 89,418,898/108,429,611 islets, 81,359,243/98,250,529 hypothalamus, and 49,297,475/61,390,433 liver were successfully aligned by employing the above criteria. BTBR/B6 raw sequence data is available at NCBI Short Read Archive under accession SRA008619.3; previously published CAST/B6 data is accessible under SRA008621.10.

### Quantification of allele-specific events from sequencing data

ASE was assessed by summing allele-specific reads that align over independently identified SNPs [14]; using the SNP to distinguish the allelic origin. For comparison with microarray transcript levels, allelic counts were summed across all SNPs within the transcript boundaries (including introns), provided that the sequencing reads were in the same orientation as the transcript. To exclude artifactual allelic bias arising from false-positive SNPs or differential priming events, we required a minimum of three SNPs within the gene and agreement on strain bias at the majority of all SNPs/transcript. We excluded SNPs where the average Illumina-phred score was below 20, since scores below 20 do not accurately reflect sequencing errors (Illumina, personal communication). We also excluded C/A, A/C, and G/T (B6/BTBR) SNPs since the sequence data had disproportional amounts of reference mismatches of these variety, indicative of high error rates.

Expected null overlap of genes under *cis*-eQTL and genes with ASE with the same directional bias was computed as

$$\left(\frac{Array\_genes>Add\_Eff}{All\_genes}\right)\left(\frac{Seq\_genes>LBP}{All\_genes}\right)\left(All\_genes\right)\left(\frac{1}{2}\right)$$

where All_genes was the total number of genes on the microarray after removal of genes with SNPs in probe regions (n = 34,257).

Antisense analysis was conducted for SNPs where reads mapped to both strands. Since our NSR-seq approach may incorrectly detect up to 0.7% antisense reads [15], we used the binomial to exclude potential artifacts where expression is predominant from one strand (p < 1e-4; Bonferroni-corrected). LBP scores were normalized to the mean for each strand (Figure 4b) to correct for overlap arising from slightly higher numbers of B6 reads than BTBR (presumably from genomic alignment bias). Allele-specific splicing was assessed when a SNP overlaps reads that support an alternative splice junction (i.e. both isoforms are represented). Only reads with the same orientation as the spliced transcript were considered.

### Experimental validation of allele-specific antisense events

0.5 ug total RNA pooled from four B6 × CAST and four CAST × B6 9.5 day-old embryos was either 1) reverse transcribed using Qiagen OneStep RT-PCR kit (Qiagen) according to the manufacturer's instructions with 35 rounds of PCR (for alternative splice-site amplification), or 2) reverse-transcribed with superscript III (Invitrogen), RNAse H treated (Invitrogen), and amplified by Roche high-Fidelity PCR (Roche; 35 cycles) according to the manufacturer's instructions (for strand-specific amplification of antisense transcription). Primer sequences are listed in Additional File 1, Table S2.

## Authors' contributions

TB carried out the analysis. PGE and TB carried out the ASE and cis-eQTL validations. CA, and CKR carried out the NSR and generated the sequencing data. MK, AA, and ES coordinated and executed the mouse crosses and assisted with the experimental design. TB, RC, CMR, JJ, AA, HF, and ES conceived of the study and wrote the manuscript. All authors read and approved the final manuscript.

## Supplementary Material

Additional file 1 **Supplemental Material**. Supplemental Figures, Tables, Methods, and Discussion Supplement Click here for file
