# Supplementary material for: Genetic validation of whole-transcriptome sequencing for mapping expression affected by cis-regulatory variation
Source: BMC Genomics. 2010 Aug 13;11:473. doi: 10.1186/1471-2164-11-473 (PMC3091669; doi:10.1186/1471-2164-11-473)
Supplement: Additional file 1 — Supplemental Material. Supplemental Figures, Tables, Methods, and Discussion Supplement [file 1471-2164-11-473-S1.PDF]

Supplementary Material **Page**

Supplementary Material	Page
Supplementary Figure Legends	2
Supplementary Figures	
Figure 1: Summary of NSR-seq data	3
Figure 2: Tissue-specificity of ASE	4
Figure 3: cis-eQTL/ASE overlap in islets	5
Figure 4: cis-eQTL/ASE overlap in F1s	6
Figure 5: ASE quantification in islets	7
Figure 6: Antisense analysis in adipose BTBRxB6 data	8
Figure 7: Supporting Sanger traces for Figure 5 in the main manuscript	9
Figure 8: Schematic for detection of allele-specific splicing	10
Supplementary Table 1: Validating RNA-seq/Arrays by Sanger-seq	11
Supplementary Table 2: RT-PCR probe sequences (antidirectional validation)	13
Supplementary Table 3: Primer sequences used for Supp. Table 1.	14
Supplementary Methods	15
Discussion Supplement	16

Supplementary Figure Legends

Figure 1
Quantification of NSR-seq parameters required for measuring allele-specific expression in the four tissues sampled: **(a)** Number of aligned sequencing reads to RefSeq genes. **(b)** Number of aligned sequencing reads to RefSeq genes that overlap a BTBR/B6 SNP and can thus be used to infer allelic origin. **(c)** Number of SNPs per RefSeq gene including introns. **(d)** Number of SNPs with at least one supporting read.

Figure 2
Pairwise ASE comparisons of four tissues. LBP scores (see text for explanation) are plotted for all RefSeq genes detected in both tissues by at least one allele-specific sequencing read (number of genes [n] indicated in plots). Significant positive correlations were observed for all combinations indicating that *cis*-acting expression effects tend to act similarly across tissues.

Figure 3
Genes under cis-eQTL (Array) and genes under allele-specific expression (ASE; RNA-seq) overlap and agree on direction of allelic bias in islets. **(a)** Ratio of observed to expected level of overlapping genes exceeding Genetic Additive Effect and ASE confidence scores (log(binomial-p); LBP). **(b)** Proportion of overlapping genes for which ASE and Additive Effect agree on direction of allelic bias (i.e. B6 vs. BTBR).

Figure 4
cis-eQTL overlap with ASE in F1 hybrids. We generated additional sequencing data from four C57Bl/6J x CAST male F1 hybrid adipose samples (pooled at RNA level by equal mass) and compared it to cis-eQTL generated from F2s in the same cross (GEO Accession: GSE16227). Here we plot the number of ASE genes (binomial $p<0.01$) that are identified for each cis-eQTL gene, rank ordered on absolute additive effect in descending order. The top 200 genes are shown.

Figure 5
Identical analysis as per Figure 3 in main text, but for islets (cf. adipose).

Figure 6
Identical analysis as per Figure 4 in main text, but for adipose BTBRxB6 data.

Figure 7
Supporting Sanger traces for Figure 4 in the main manuscript.

Figure 8
Schematic for detection of allele-specific splicing.

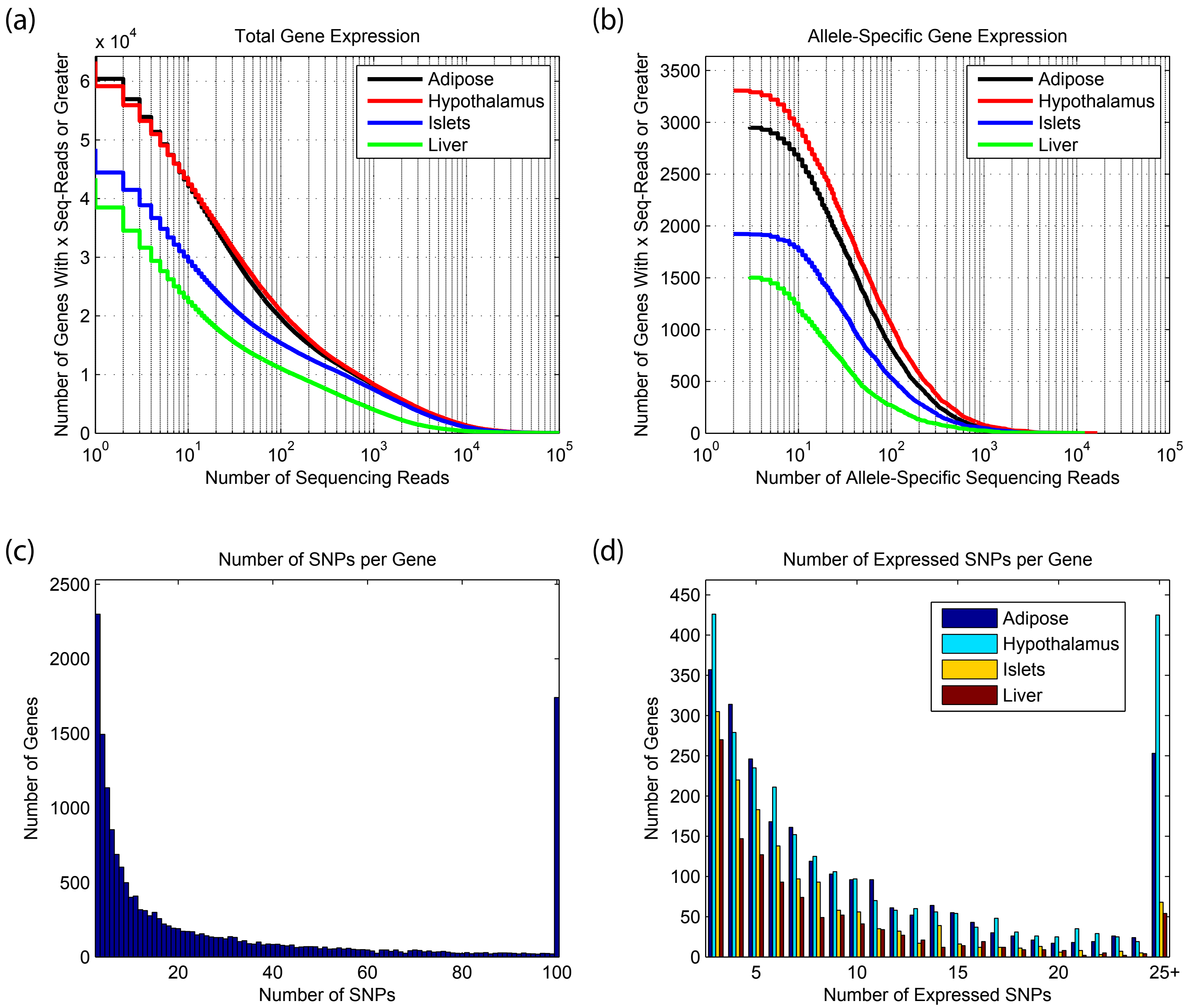

Supp Figure 1

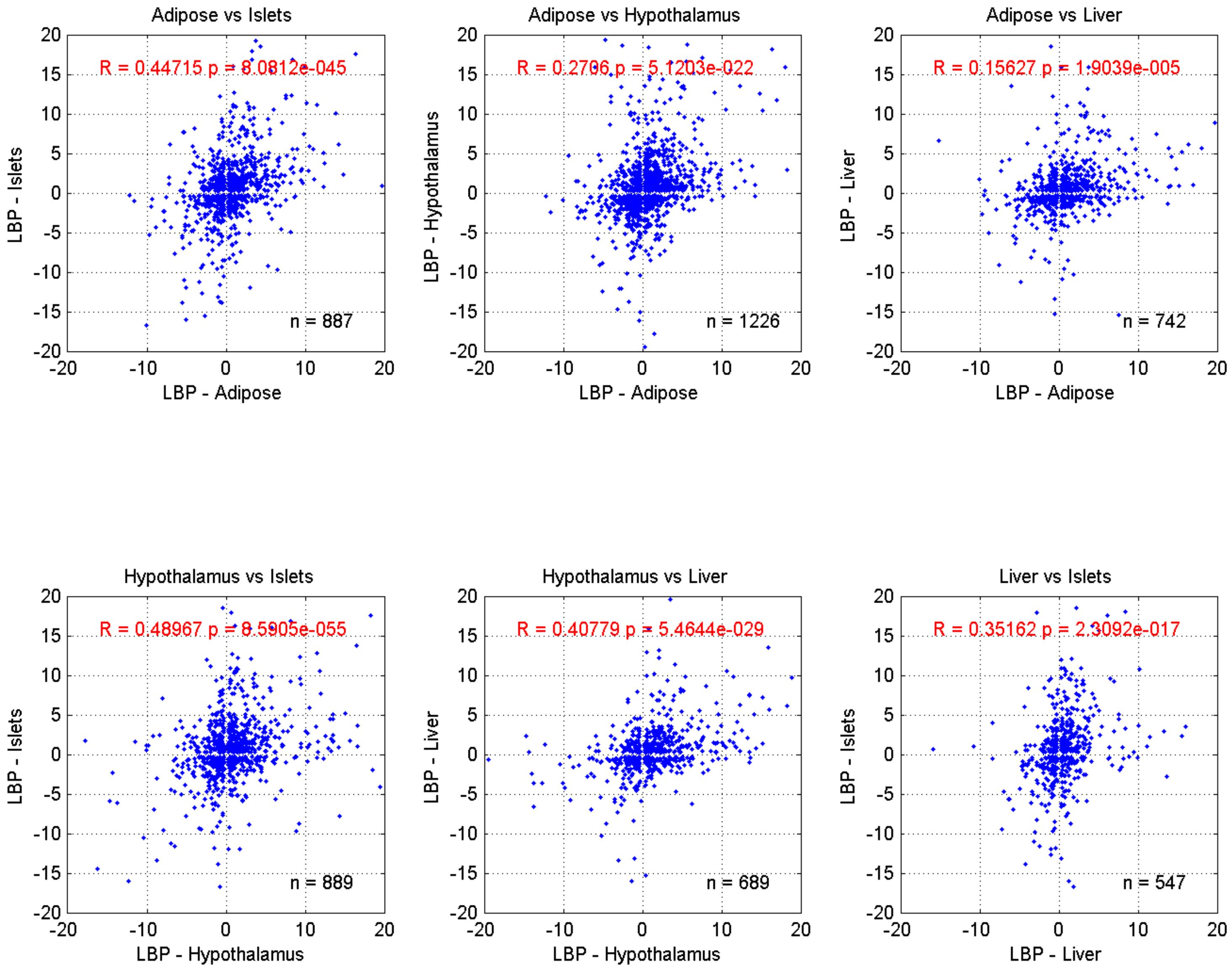

Supp Figure 2

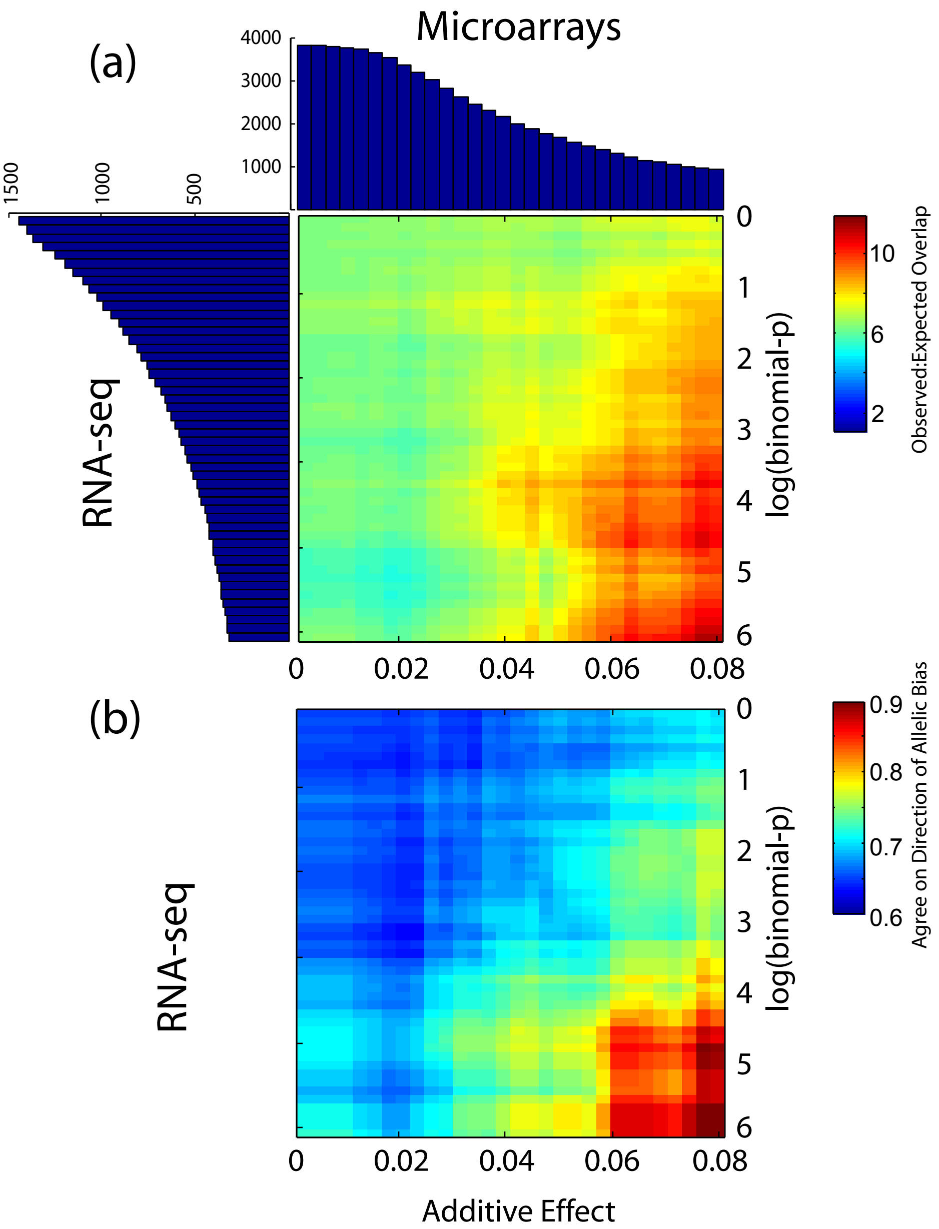

Supp Figure 3

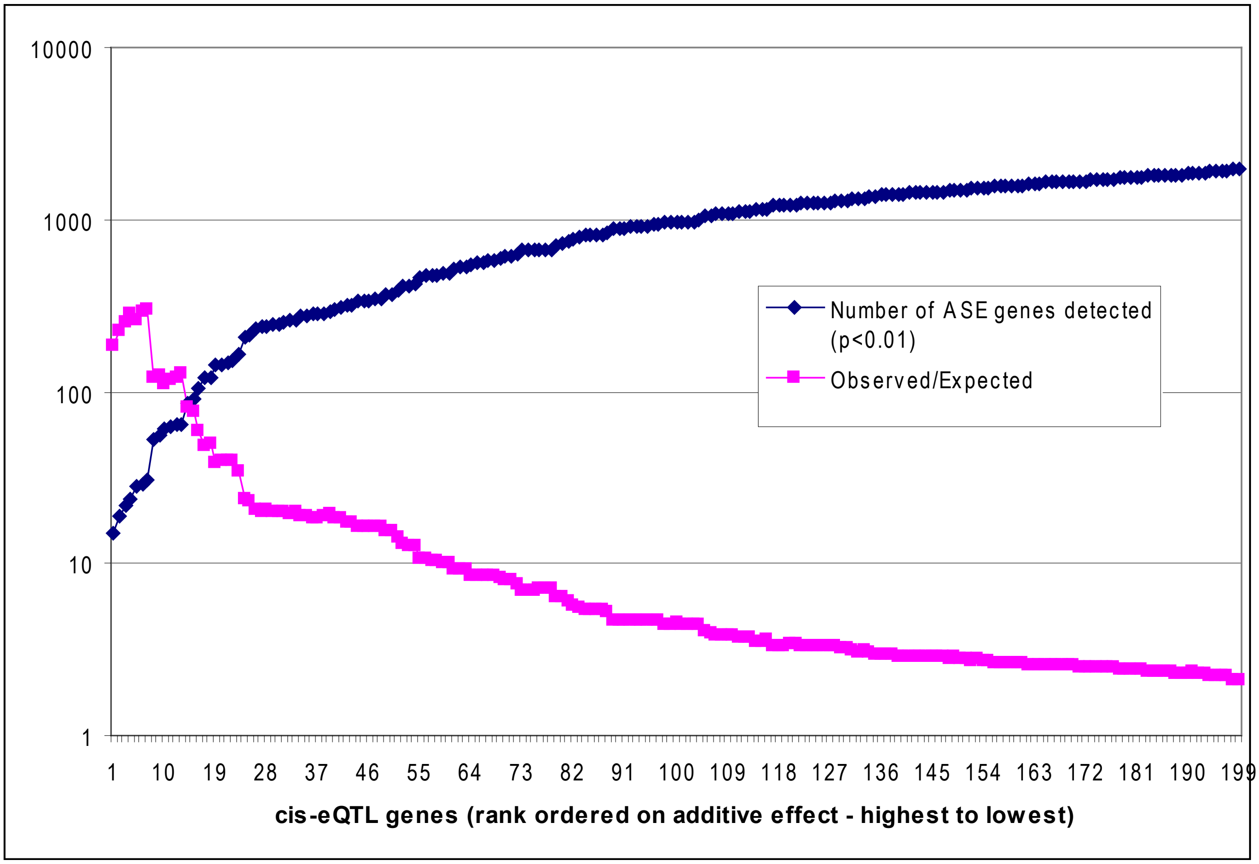

Supp Figure 4

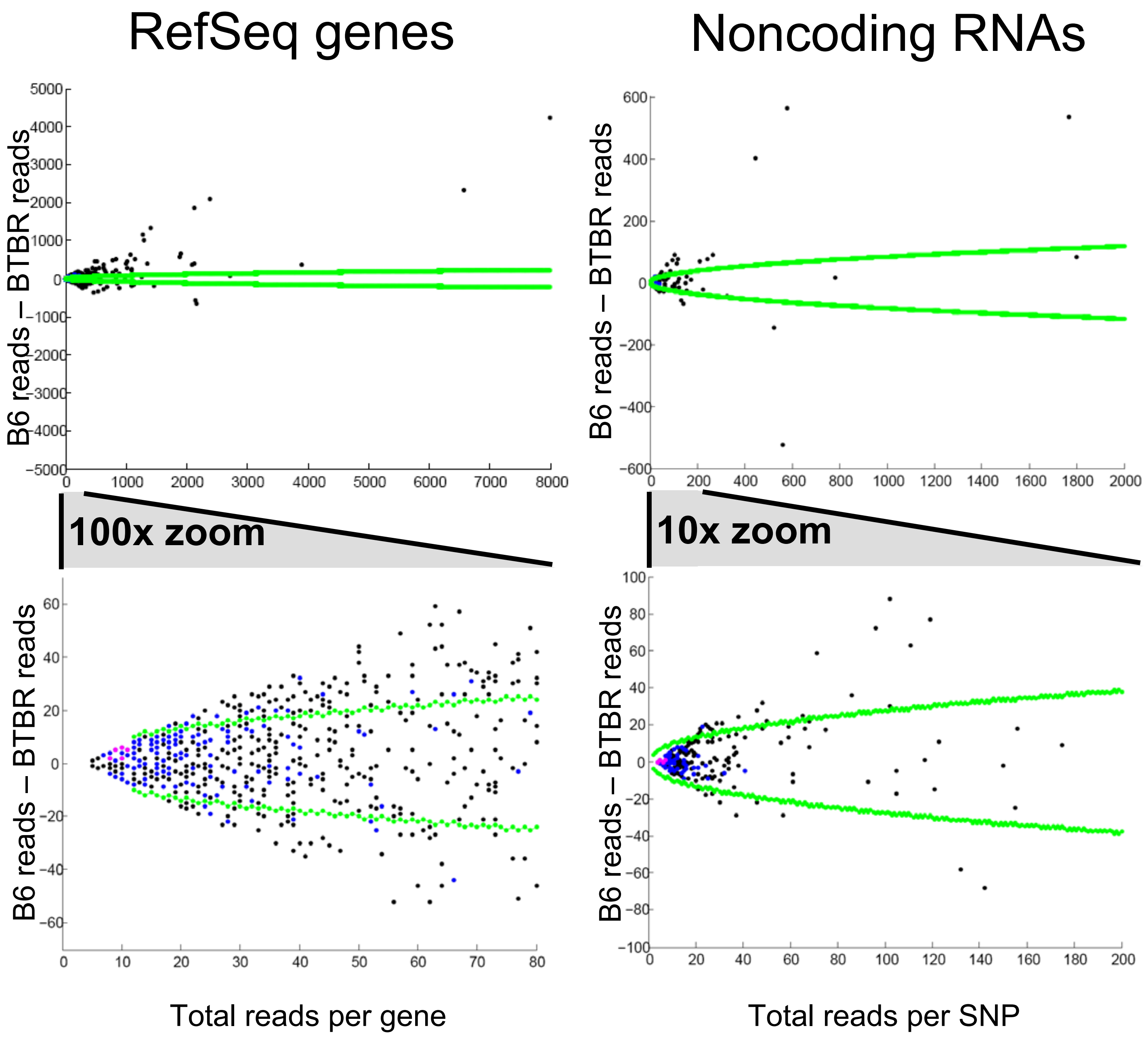

Supp Figure 5

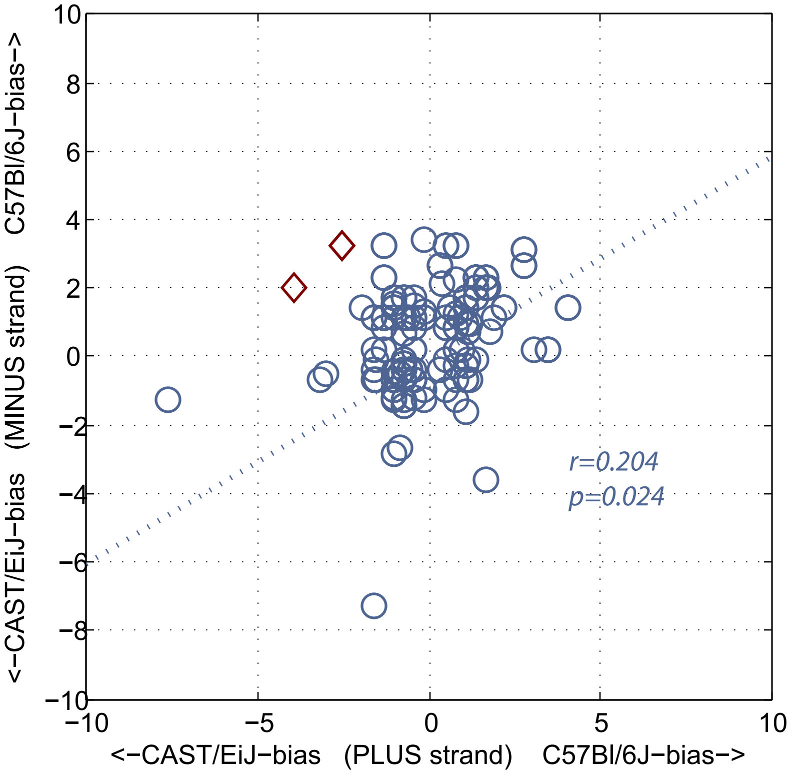

Supp Figure 6

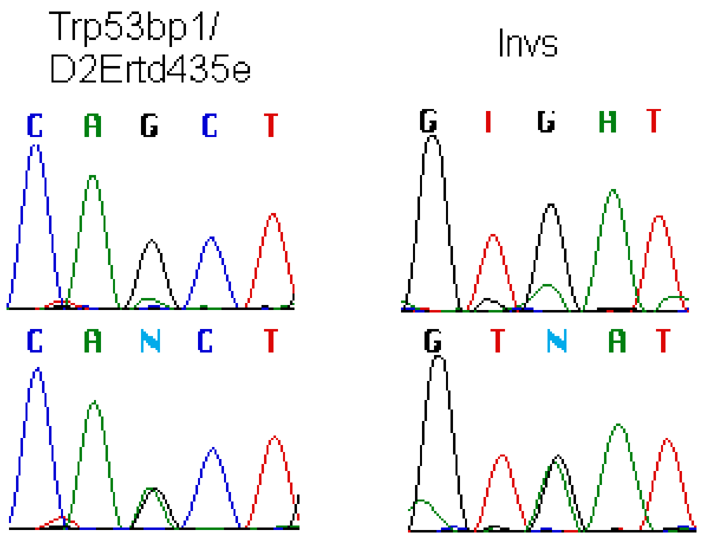

Supp Figure 7

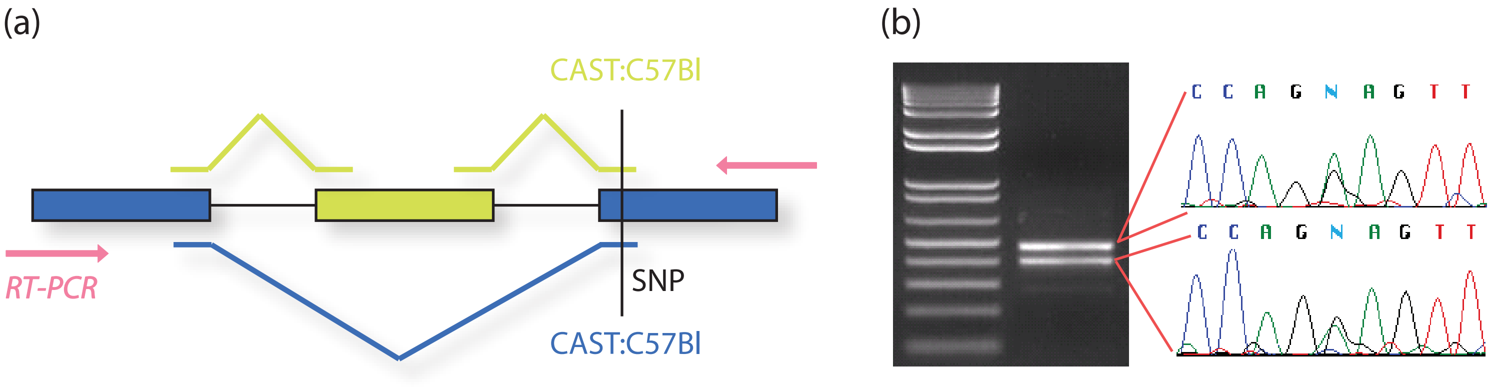

Supp Figure 8

Table 1. Summary of Sanger sequencing RT-PCR products from adipose F2 pool that capture genes where microarrays and RNA-seq disagree on direction of bias (B6 vs. BTBR). Each product captures one SNP that was selected on maximum RNA-seq sequencing depth. Please see Supplementary File 1 for all Sanger traces. See supplementary Table 3 for primer sequences used herein.

<table>
<tr><th>Assay ID</th><th>Gene</th><th>LOD</th><th>Add. Eff.</th><th>Seq Data (B6|BTBR; one netry per SNP in gene)</th><th>Most abundant SNP (B6|BTBR)</th><th>SNP-BED</th><th>SNP</th><th>Sanger</th><th>Supp File</th></tr>
<tr><td>ID01</td><td>D17Wsu92e</td><td>44.89</td><td>-0.327462202</td><td>5|11,0|4,3|0,212|119,4|0</td><td>212|119</td><td>chr17:27513822</td><td>T|C</td><td>FP SNP</td><td>dis19</td></tr>
<tr><td>ID02</td><td>Gprc5b</td><td>37.83</td><td>-0.15787974</td><td>3|0,1|2,49|34,3|0,5|0,38|24,8|0</td><td>49|34</td><td>chr7:118765435</td><td>C|T</td><td>No Call</td><td>dis5</td></tr>
<tr><td>ID03</td><td>Bckdhb</td><td>46.64</td><td>-0.138004575</td><td>5|0,8|0,0|1</td><td>8|0</td><td>chr9:83890051</td><td>G|C</td><td>FP SNP</td><td>dis11</td></tr>
<tr><td>ID04</td><td>Zmat3</td><td>49.14</td><td>-0.127577048</td><td>5|1,3|4,20|0,8|3,3|0,1|0,1|0,0|2</td><td>20|0</td><td>chr3:32531439</td><td>G|A</td><td>Agree with RNA-seq</td><td>dis7</td></tr>
<tr><td>ID05</td><td>AI450540</td><td>42.52</td><td>-0.111342467</td><td>4|0,2|0,2|0,0|1,4|0,3|0,60|20,24|12,18|14,0|3,5|0</td><td>60|20</td><td>chr19:57435048</td><td>T|C</td><td>Agree with RNA-seq</td><td>dis20</td></tr>
<tr><td>ID06</td><td>Adi1</td><td>25.84</td><td>-9.03E-02</td><td>100|0,4|0,2|11,18|0,0|1</td><td>100|0</td><td>chr12:29265812</td><td>T|C</td><td>FP SNP</td><td>dis16</td></tr>
<tr><td>ID07</td><td>Wtap</td><td>4.92</td><td>-7.99E-02</td><td>2|0,140|0,0|0</td><td>140|0</td><td>chr17:12823777</td><td>A|G</td><td>No Call</td><td>dis17</td></tr>
<tr><td>ID08</td><td>Mcpt4</td><td>7.37</td><td>-7.27E-02</td><td>118|0,133|0,7|3</td><td>133|0</td><td>chr14:55014115</td><td>T|G</td><td>Agree with RNA-seq</td><td>dis1</td></tr>
<tr><td>ID09</td><td>Epn2</td><td>9.59</td><td>-6.60E-02</td><td>0|2,7|0,0|2,19|8,2|0,2|0</td><td>19|8</td><td>chr11:61347991</td><td>A|G</td><td>FP SNP</td><td>dis8</td></tr>
<tr><td>ID10</td><td>Hebp1</td><td>11.04</td><td>-6.51E-02</td><td>11|2,23|10,16|5,2|2,1|6,3|0,0|1</td><td>23|10</td><td>chr6:135103653</td><td>A|G</td><td>Agree with RNA-seq</td><td>dis15</td></tr>
<tr><td>ID11</td><td>Ube4a</td><td>25.10</td><td>-6.29E-02</td><td>3|0,2|0,2|0,4|0,0|3,0|5,0|4,11|0,3|0,3|2,12|0,1|0,24|20,1|0</td><td>24|20</td><td>chr9:44715308</td><td>A|T</td><td>Agree with RNA-seq</td><td>dis25</td></tr>
<tr><td>ID12</td><td>Pot1b</td><td>22.14</td><td>-6.16E-02</td><td>1|0,1|1,4|0,0|1,1|0,0|3,26|2,1|0,1|0</td><td>26|2</td><td>chr17:55291496</td><td>T|C</td><td>Agree with RNA-seq</td><td>dis22</td></tr>
<tr><td>ID13</td><td>Cd2ap</td><td>10.97</td><td>-0.061501604</td><td>286|2,15|0,1|0,1|3,0|1,0|2,0|8,6|0,39|5</td><td>286|2</td><td>chr17:42259004</td><td>C|T</td><td>Agree with RNA-seq</td><td>dis14</td></tr>
<tr><td>ID14</td><td>Foxn3</td><td>12.61</td><td>-5.73E-02</td><td>28|20,1|2,8|4,61|38,4|0,0|1,2|0,5|2,23|12,5|0,0|1,3|0,2|2,5|0,29|24,3|0,0|4,1|0,0|1,1|0,1|0,5|0,9|4,1|1,…</td><td>61|38</td><td>chr12:99616985</td><td>G|A</td><td>No Call</td><td>dis10</td></tr>
<tr><td>ID15</td><td>Snx19</td><td>29.98</td><td>-5.61E-02</td><td>3|0,0|2,1|0,1|0,16|0,3|0,2|0,0|3,2|0,1|0,0|2,16|18</td><td>16|18</td><td>chr9:30215997</td><td>G|A</td><td>Agree with RNA-seq</td><td>dis26</td></tr>
<tr><td>ID16</td><td>4931406I20Rik</td><td>13.12</td><td>-5.35E-02</td><td>2|0,3|0,8|0,0|2,3|0,2|0,15|0,0|1,2|0,2|0,3|0,2|0,5|0,0|2</td><td>15|0</td><td>chr4:116888651</td><td>T|C</td><td>No Call</td><td>dis9</td></tr>
<tr><td>ID17</td><td>4930579E17Rik</td><td>10.82</td><td>-5.18E-02</td><td>2|0,0|4,2|0,3|0,0|2,8|1,0|1,2|0,2|0,8|0</td><td>8|1</td><td>chr12:37060649</td><td>T|C</td><td>FP SNP</td><td>dis2</td></tr>
<tr><td>ID18</td><td>Vps13d</td><td>15.43</td><td>5.32E-02</td><td>12|13,8|3,5|11,2|8,0|1,0|2,13|11,5|3,0|3,1|0,3|0,2|0,0|3,0|3,2|1,6|2,0|1,0|3,0|1,1|0,1|0,0|1,0|3,2|0,5|0,51|38…</td><td>63|160</td><td>chr4:144434847</td><td>G|C</td><td>No Call</td><td>dis23</td></tr>
<tr><td>ID19</td><td>Rchy1</td><td>29.85</td><td>5.54E-02</td><td>0|1,0|2,0|6</td><td>0|6</td><td>chr5:93032269</td><td>C|G</td><td>No Call</td><td>dis27</td></tr>
<tr><td>ID20</td><td>Abca4</td><td>6.06</td><td>0.055975888</td><td>3|0,0|3,0|2,0|10</td><td>0|10</td><td>chr3:122154077</td><td>A|G</td><td>Agree with microarrays</td><td>dis4</td></tr>
<tr><td>ID21</td><td>Cd200</td><td>3.82</td><td>5.97E-02</td><td>5|10,24|99,0|7,1|0,2|3,9|1,11|3,3|5</td><td>24|99</td><td>chr16:45313824</td><td>C|T</td><td>Agree with RNA-seq</td><td>dis24</td></tr>
<tr><td>ID22</td><td>Zfp68</td><td>32.41</td><td>9.11E-02</td><td>0|1,14|32,80|117,6|1,8|2,2|0,0|2,0|2,1|6,1|0</td><td>80|117</td><td>chr5:138837023</td><td>A|G</td><td>No Call</td><td>dis6</td></tr>
<tr><td>ID23</td><td>Stk25</td><td>54.70</td><td>0.172987184</td><td>0|19,1|4,0|1</td><td>0|19</td><td>chr1:95454412</td><td>T|A</td><td>Agree with RNA-seq</td><td>dis12</td></tr>
<tr><td>ID24</td><td>Trps1</td><td>63.22</td><td>0.219227804</td><td>27|70,3|0,31|15,0|4,1|3,0|1,2|0,1|1,0|3,1|0,2|0,2|0,0|4,28|35,1|9,0|5,14|4,0|1,3|8</td><td>27|70</td><td>chr15:50491010</td><td>C|T</td><td>Agree with microarrays</td><td>dis21</td></tr>
<tr><td>ID25</td><td>Ndufaf2</td><td>70.25</td><td>0.393522858</td><td>4|30,0|2,8|5,4|8</td><td>4|30</td><td>chr13:109173543</td><td>C|T</td><td>Agree with microarrays</td><td>dis3</td></tr>
</table>

Supplementary Table 2. Primer sequences used for validating allele-specific antidirectional transcription and RNA-seq counts used for identification.

ID	SNP	fwd primer	rev primer	B6 counts (+ strand)	CAST counts (+ strand)	B6 counts (- strand)	CAST counts (- strand)
Phactr4	chr4:131637354	GAAGTTAACTATGTACAGGGATAGGAA	CCTGTGTGTGATATGCCAGAA	17	0	0	17
Ptprd	chr4:75473800	GCATCTTCTTCCTCTGGGACT	GATCTAGGCTGAGGGTTTCCA	14	0	0	12
Trp53bp1	chr2:120889369	CCGCTGGCTTTATTTAGAGA	TTCCAAAAACAGTTTAAAGTAGGTGA	23	5	33	87
Invs	chr4:48451236	CTGGGCTGGGATTTCTAACA	CTCTCTGGACCAACCTGGAA	1	5	9	0

Supplementary Table 3. Primer sequences used for validation.

Assay	Gene/ID	FWD primer	REV primer
Antisense validation	Phactr4	GAAGTTAACTATGTACAGGGATAGGAA	CCTGTGTGTGATATGCCAGAA
Antisense validation	Ptprd	GCATCTTCTTCCTCTGGGACT	GATCTAGGCTGAGGGTTTCCA
Antisense validation	Trp53bp1	CCGCTGGCTTTATTTAGAGA	TTCCAAAAACAGTTTAAAGTAGGTGA
Antisense validation	Invs	CTGGGCTGGGATTTCTAACA	CTCTCTGGACCAACCTGGAA
RNA-seq/Array Validation	ID01	AGTGCACATCCGCCACTGT	CCTCCAGGGGTTTGTCTTAAA
RNA-seq/Array Validation	ID02	CAGGATGCCATCTCTCGAT	AGAAATATCCCTCCTCCTTCCTC
RNA-seq/Array Validation	ID03	ATGGGAGGACTCTGTGTTCC	GACAGTCACCAGCAGCTTCA
RNA-seq/Array Validation	ID04	GCAACCAAATGGCTAACAAGA	ATGGAGAGGGAGGAAATGCT
RNA-seq/Array Validation	ID05	AGCATCATGACCCTGAGGA	CCTGCTATCAGCCCGTTCT
RNA-seq/Array Validation	ID06	TGTTCTTTGAGGAACATCTGC	CTTCTCGTCCAGTGTGAAGC
RNA-seq/Array Validation	ID07	GGTGCACTCTTGCATCTCCT	TGTGACTGGTTTAAGGGAATCTG
RNA-seq/Array Validation	ID08	TGGCTTGAAGACTCTGATGC	TGGAGGACCTCTACTGTGTGC
RNA-seq/Array Validation	ID09	TTCCCCTCACCCTTCTACCT	GCTAGGCTCTGTCCTCCCATA
RNA-seq/Array Validation	ID10	CCCTAAGTCAGGGGACACAG	GCTATGCCAAGGAAGCAGAC
RNA-seq/Array Validation	ID11	GCCACACAGAAACACATCCA	TGTATGCTCTCAGGAGCCAGT
RNA-seq/Array Validation	ID12	TGGATCTAGTCCCCATCACAG	GATACCCTTCCCAGCGTTTA
RNA-seq/Array Validation	ID13	TCTGGGTTTAGGTTTGTTTCC	GGATTAATCTTGAGAAAATAGGAGCTT
RNA-seq/Array Validation	ID14	AGGACATATACCCCGGTTCC	AAAACGTGGTGGTGTAGACG
RNA-seq/Array Validation	ID15	TCCACCTGCCTTCAAAAGAT	GGGTGTTGGTCTTACTTCAGG
RNA-seq/Array Validation	ID16	TTCTAAGGTGGAGGCATTTCA	GCTGTGTCATCCACTGATGG
RNA-seq/Array Validation	ID17	GGTGTGAGCAATCAGCAAAA	TTTTAGAAGCAAAGCTACATTGAA
RNA-seq/Array Validation	ID18	TCTGCACAAAGAGCAGGATG	GAACCGCAGTTTGATTCTCC
RNA-seq/Array Validation	ID19	AGAATCACTGTGCTGAACTCCA	ACAGGAGACCCAGTGTGCAG
RNA-seq/Array Validation	ID20	GCCGTAGAGCAACGGTTTTA	TCCTCTGGACTTCTTTTCTCTAAGTC
RNA-seq/Array Validation	ID21	GGTTCCATACTTTCTGCAGGTT	CTGGGGAATGTGATTGACTACA
RNA-seq/Array Validation	ID22	CCTCTGCGGATTTTCTGATG	TGGCTCAGATAAGGGTGAAA
RNA-seq/Array Validation	ID23	CCCTAAGGAGTCATCTGCTCA	ATACAGTGCGGCCAGGAAT
RNA-seq/Array Validation	ID24	GCATATGCTGCACTGGAAAG	AGATTTGGCGATCAAGCATT
RNA-seq/Array Validation	ID25	TCATTCATTGACTTTGTCATTCAT	AAACTCGGTAAAGAAACCAGTGA

Supplementary Methods

Islet isolation

Islets were carefully hand-picked under a stereo microscope to remove contaminating acinar tissue, after which the islets were washed twice with phosphate buffered saline (PBS) and centrifuged at 2,500 rpm, 5 minutes, 4°C after which PBS supernatant was removed. Islets were homogenized by hand for 1 minute with a plastic micro-pestel (USA Scientific) in RLT buffer (Qiagen), and stored at -80°C until RNA purification. RNA was purified using the Qiagen RNeasy Mini Kit, according to manufacturer directions and integrity was verified on an Agilent Bioanalyzer 2100. All animal handling procedures were approved by University of Wisconsin Animal Care and Use Committee.

RNA isolation and microarray analysis

RNA preparation for adipose tissue and all array hybridizations were performed at Rosetta Inpharmatics (Merck & Co., Seattle, WA, USA). The custom ink-jet microarrays used in this study were manufactured by Agilent Technologies (Palo Alto, CA) and consisted of 4,732 control probes and 35,555 non-control oligonucleotides extracted from mouse Unigene clusters and combined with RefSeq sequences and RIKEN full-length cDNA clones. Mouse tissues were homogenized and total RNA extracted using Trizol (Invitrogen, CA, USA) according to manufacturer's protocol. Total RNA was reverse transcribed and labeled with either Cy3 or Cy5 flurochrome. A reference pool of RNA for each tissue was constructed by pooling equal aliquots of RNA from 100 randomly selected, gender balanced F2 mice. cRNA from each F2 animal was hybridized against the tissue-specific pool for each tissue. All hybridizations were performed for 48 hours in a hybridization chamber, washed, and scanned using a confocal laser scanner. Arrays were quantified on the basis of spot intensity relative to background, adjusted for experimental variation between arrays using average intensity over multiple channels, and fitted to a previously described error model to determine significance (type I error) [1]. Gene expression measures are reported as the ratio of the mean log10 intensity (mlratio). 1,061 genes with probes that overlap a previously reported BTBR/B6 SNP [2] and 237 probes that overlap SNP predictions generated from sequencing reads (as previously described) [3] were excluded from analysis.

RNA amplification

Strand-specific, whole-cell (total RNA) RNA-seq: Please see Armour et al [4] for details. In brief: 1 ug total RNA from each sample was reverse-transcribed with Superscript III (Invitrogen) in a 20 uL reaction containing 2 mM dNTP, 5 mM DTT, 1 unit RNAse OUT, and 10 uM primers Armour et al. (2009) for sequences, incubated 40°C for 60 minutes, 70°C for 15 minutes, and cooled to 4°C. RNA was degraded by addition of 1 uL RNAse H (Invitrogen) and incubated at 37°C for 20 minutes then heat-denatured at 75°C for 15 minutes. A longer incubation time from that recommended by Armour et al (2009) was used to improve transcript coverage. Following PCR

purification (Qiagen), first-strand product was subjected to second strand synthesis in a 100 uL reaction containing 10 uM antisense primers, 16.5 units Klenow (NEB: M0212L), and 0.2 mM dNTP by incubating 37°C for 30 minutes. Following PCR-purification (Qiagen), Illumina-specific adaptors and priming sites were added through PCR (see Supplementary Table 1 for sequences) using Roche High-Fidelity system at recommended conditions for 35 cycles (94°C 15", 60°C 30", 72°C 1'+10"/cycle). To ensure initial primer annealing, annealing temperature for the first two cycles was reduced to 40°C for 2 minutes. Following PCR purification (Qiagen), library integrity was verified by resolution on 2% agarose.

Discussion Supplement

"compiling catalogs of genes affected by cis-eQTL in various tissues, populations, and disease states could be extremely useful in this regard"… our method does not actually pinpoint the causal SNPs responsible for the cis-eQTL, but it will implicate a responsible haplotype (whose full extent can be measured by considering LD structure from the HapMap). By searching for overlaps between those cis-eQTL haplotypes and SNPs implicated by GWAS, we can generate lists of disease associations likely to be mediated by cis-effects on gene expression, as well as the affected genes—reconciling some of the subjectivity that currently pervades GWAS candidate gene lists.

1. He YD, Dai H, Schadt EE, Cavet G, Edwards SW, Stepaniants SB, Duenwald S, Kleinhanz R, Jones AR, Shoemaker DD *et al*: **Microarray standard data set and figures of merit for comparing data processing methods and experiment designs**. *Bioinformatics (Oxford, England)* 2003, **19**(8):956-965.
2. Frazer KA, Eskin E, Kang HM, Bogue MA, Hinds DA, Beilharz EJ, Gupta RV, Montgomery J, Morenzoni MM, Nilsen GB *et al*: **A sequence-based variation map of 8.27 million SNPs in inbred mouse strains**. *Nature* 2007, **448**(7157):1050-1053.
3. Babak T, Deveale B, Armour C, Raymond C, Cleary MA, van der Kooy D, Johnson JM, Lim LP: **Global survey of genomic imprinting by transcriptome sequencing**. *Curr Biol* 2008, **18**(22):1735-1741.
4. Armour CD, Castle JC, Chen R, Babak T, Loerch P, Jackson S, Shah JK, Dey J, Rohl CA, Johnson JM *et al*: **Digital transcriptome profiling using selective hexamer priming for cDNA synthesis**. *Nat Methods* 2009.
